# CoCoMac 2.0 and the future of tract-tracing databases

**DOI:** 10.3389/fninf.2012.00030

**Published:** 2012-12-27

**Authors:** Rembrandt Bakker, Thomas Wachtler, Markus Diesmann

**Affiliations:** ^1^Donders Institute for Brain, Cognition and Behaviour, Radboud University NijmegenNijmegen, Netherlands; ^2^Institute of Neuroscience and Medicine 6, Research Center JülichJülich, Germany; ^3^Department Biology II, Ludwig-Maximilians-Universität MünchenMunich, Germany; ^4^Faculty of Medicine, RWTH Aachen UniversityAachen, Germany; ^5^RIKEN Brain Science InstituteWako, Saitama, Japan

**Keywords:** CoCoMac, macaque, connectivity, database, axonal tracing

## Abstract

The CoCoMac database contains the results of several hundred published axonal tract-tracing studies in the macaque monkey brain. The combined results are used for constructing the macaque macro-connectome. Here we discuss the redevelopment of CoCoMac and compare it to six connectome-related projects: two online resources that provide full access to raw tracing data in rodents, a connectome viewer for advanced 3D graphics, a partial but highly detailed rat connectome, a brain data management system that generates custom connectivity matrices, and a software package that covers the complete pipeline from connectivity data to large-scale brain simulations. The second edition of CoCoMac features many enhancements over the original. For example, a search wizard is provided for full access to all tables and their nested dependencies. Connectivity matrices can be computed on demand in a user-selected nomenclature. A new data entry system is available as a preview, and is to become a generic solution for community-driven data entry in manually collated databases. We conclude with the question whether neuronal tracing will remain the gold standard to uncover the wiring of brains, thereby highlighting developments in human connectome construction, tracer substances, polarized light imaging, and serial block-face scanning electron microscopy.

## INTRODUCTION

The Frontiers in Neuroinformatics Special Topic “Mapping the Connectome” is dedicated to the memory of Rolf Kötter, founding father of the Macaque connectivity database CoCoMac (Stephan et al., 2001; Kötter, 2004). This database contains the results of about 300 published axonal tract-tracing studies, and another 150 studies on brain atlases and nomenclature. In the last 3 years of his life, while continuously stepping up the fight against the disease that threatened his life, Rolf Kötter energetically led an effort to improve the informatics and databasing aspects of the CoCoMac database. After his tragic death in 2010, we have stepped forward to keep this ongoing project alive. We decided to keep the CoCoMac website cocomac.org in a “frozen” but functional state and refer to it as CoCoMac 1.0. The newly developed CoCoMac 2.0 is hosted at cocomac.g-node.org.

In this article we present the status of CoCoMac 2.0, and compare its neuroinformatics aspects to six connectome-related projects that have recently been introduced or updated; the names in ***bold italics*** are used to reference the project throughout this paper:

1. The rat ***temporal-lobe project*** of Sugar et al. (2011). This project covers a limited part of the brain, but maximizes the level of detail that can be obtained from published tracing studies.
2. The Brain Architecture Management System (***BAMS***) of Bota et al. (2012), a well-established system for storage, retrieval, and searching of connectivity data at all levels of detail, largely populated with rat tract-tracing data, but also connected to a new ***mouse connectome***^1^ initiative.
3. The ***Connectome Viewer*** Toolkit (Gerhard et al., 2011), which provides advanced surface, volume, and graph rendering tools in a Python package.
4. The rat connectivity component of the ***rodent brain workbench*** (Zakiewicz et al., 2011), providing public access to raw tract-tracing images, registered to a common atlas space.
5. The ***Allen mouse connectivity atlas***^2^, with open access to raw data of viral, cell type-specific tracers.
6. The ***neuroVIISAS*** rat connectome system of Schmitt and Eipert (2012), a software package that covers the entire pipeline from connectivity database and atlasing to brain simulations

The organization of this paper is as follows: we briefly present the history of CoCoMac and discuss its unique features; we then present components of the new CoCoMac and a longer term perspective. Along the way, we compare the various informatics aspects to the six connectome-related projects.

## CoCoMac 1.0

CoCoMac 1.0 started in the late 1990s as an MS Access (Microsoft Corporation, WA, USA) database, to collect data from tract-tracing studies in the macaque brain. Although the research questions in these studies vary widely, they have in common that injections are done in the brain, and the location and density of labeled axon terminals (anterograde tracer) or cell bodies (retrograde tracer) are observed. The biggest challenge is to accurately capture *where* in the brain the injections and labeled sites are. Macaque tracing studies almost invariably use a *brain map* that parcellates the brain into named regions, based on cytoarchitectonic criteria (Kötter and Wanke, 2005). The tracing result is summarized in textual statements such as “the injection was in region A, with spillover to region B” and “dense labeling was observed in region C.” These textual statements degrade the tracing result, with its cellular level detail, to a macroscopic level description.

While populating the database, it was quickly discovered that in many cases, a tracing study adapts an existing brain map into a modified version. To combine tracing statements that use competing and evolving brain maps, the spatial relations between newly defined brain regions and older definitions must be known. In CoCoMac these relations are called *inter-map-relations*. They are usually provided by the paper that describes the modified brain map.

From the injections, labeled-sites, and inter-map-relations we can *compute* a macro-connectome: the matrix that describes the connection strength from each region in a given brain map to all regions in that same map. The computations involve the propagation of tracing data from its original brain map to the user-selected brain map. The objective relational transformation (ORT) algorithm by Stephan et al. (2000) pioneered this procedure. ORT uses one additional piece of knowledge: to what *extent* is the brain region covered by labeled sites (complete, partial, or none). This allows ORT to make inferences such as: “if region A has complete coverage of labeled sites, and B is a subregion of A, then also B has complete coverage of labeled sites.”

CoCoMac 1.0 contains several visionary aspects that have helped it to become the largest resource of its kind. First it does not store any type of data that requires expert interpretation. Second, it does contain an abundance of *precision of description code* (PDC) statements that answer questions such as “How well does a paper describe the location of injection X?” with possible answers “only in text,” “only in a figure,” and so on. Whenever a case of conflicting data occurs, PDC statements can be used to give some data more importance than others.

### DATA ENTRY, SEARCH, AND DISPLAY

Data entry in CoCoMac 1.0 is done in MS Access. This approach is not suitable for online data entry. The entire ORT algorithm is implemented in Visual Basic (VB) script, with pieces in Java. Performance was not an issue in the early days of CoCoMac, but now that it contains over 8000 brain sites, the ORT-based computation of a connectivity matrix takes months on a PC, due to highly inefficient code.

Visual Basic scripts also form the basis of the CoCoMac.org website. One can search for literature, inter-map-relations, and connectivity using a variety of preset criteria, and retrieve results in the form of expandable HTML tables, or XML that adheres to a published XML schema: http://cocomac.org/www/cocomac.xsd. The XML service has been used to embed connectivity data into other neuroinformatics resources including the Neuroscience Information Framework (Gardner et al., 2008), SumsDB (Dickson et al., 2001), and BrainInfo.org (Bowden et al., 2012).

The latest addition to CoCoMac 1.0 is the CoCoMac-Paxinos-3D viewer (Bezgin et al., 2009). This Java-based tool shows CoCoMac connectivity in a 3D rendering of a Macaque brain, parcellated according to the Paxinos et al. (2000) atlas.

### LESSONS LEARNED

CoCoMac 1.0 has grown to become a well-established resource, the largest of its kind, cited in 140 research articles (Google Scholar citations of Stephan et al., 2001). However, its further expansion and usage are severely limited: search functionality is restricted to three use cases; ORT calculations by the current implementation are prohibitively slow; data-entry can only be done internally.

These considerations led to the decision in 2007 that CoCoMac should be rebuilt upon an open source database engine (MySQL^3^) and scripting language (PHP^4^, along with an efficient ORT implementation, a web-based data entry system, and an online graphical display of the results.

## CoCoMac 2.0 vs. OTHER CONNECTOME PROJECTS

Here we discuss the new features of the CoCoMac 2.0 system and how they compare to the six connectome-related projects mentioned in the introduction.

### DATA ENTRY

Ideally, the data entry system not only changes the database contents, but also tracks who made the change, when, and why. We have developed a data entry system that presents each publication in CoCoMac as a hierarchical tree where nodes can be added and edited (Bakker et al., 2008). Every submitted form is stored in a table which contains all information to recreate the CoCoMac database from time zero to any desired instance of time. A view-only version of this system is available at http://cocomac.g-node.org/dataentry2010. A user management strategy with clearly defined user roles and permissions is under development.

None of the other connectome projects provides online, community-driven data entry as of today. Contributions to *BAMS* and *neuroVIISAS* must be submitted by spreadsheet. The temporal-lobe project uses MS Access internally and does not expose the database to the web. The Allen *mouse connectivity atlas* is populated by automated pipelines. The *Connectome Viewer* provides a standard file format to disseminate connectivity data. This format is a good candidate for data sharing among connectome projects. It stores the connectome as a graph, where each node typically represents a brain region, and can be assigned user-defined attributes.

It is surprising that a generic process as “web-based data entry with full provenance” is not readily available as an open source project. Besides the CoCoMac initiative, several approaches are under way in the context of lab automation, e.g., Grewe et al. (2011).

### DATA MINING: THE CoCoMac SEARCH WIZARD

With data mining we here refer to the extraction of relevant data from a single connectome database. A different branch of data mining is text mining, which could potentially replace the human data collator. However, given the key role that figures play in published tracing studies, we only see this work if authors of tracing studies provide results in a structured markup language.

**FIGURE 1:**
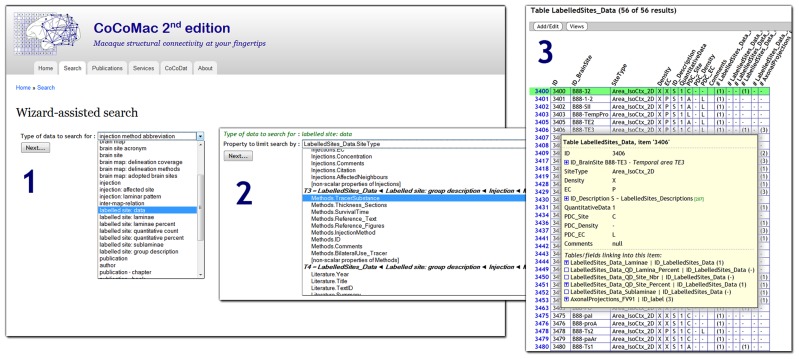
**Screenshot of the CoCoMac 2.0 search wizard (http://cocomac.g-node.org/search_wizard), with three steps highlighted: (1) selection of the data type (any database table); (2) selection of search criterion (field from table that is recursively related to data type); (3) interactive result table, expanding the tree shows all related data**.

CoCoMac 2.0 exposes its underlying database to the public, and aims to be fully open access. At the most basic level there is an SQL query interface: http://cocomac.g-node.org/sql_query. More user oriented is the *interactive query wizard* that generates complex SQL queries based on a series of intuitive search criteria. **Figure 1** shows a screenshot of the wizard^5^. The wizard is not specific to CoCoMac, but can be applied to *any* relational database. It automatically discovers its structure and foreign key relations (Data Sheet 1 in Supplementary Material).

In contrast to CoCoMac 1.0, the new search wizard does not define any use cases, but rather makes *every* table searchable. It provides filters for all properties of the table and its nested relatives. On the output side, the wizard returns data in a JSON format, which is converted to an interactive web page by client-side JavaScript. Among the other connectome projects, *BAMS* is the one with the most extensive search system. The various modules of the system contain custom search components and tabular output. As in CoCoMac, connectome matrices can be generated dynamically and provide links to the underlying literature sources. The *temporal-lobe* connectome only allows searching for connections.

### CONNECTOME GENERATION

Connectome generation lies at the heart of a connectome project. Methods that retrieve a full connectome from a single brain clearly have an advantage: they do not need to merge data from different brains, parcellations, and nomenclatures. Three such methods are discussed in the concluding section.

The *rodent work bench*, *Allen mouse connectivity atlas, *and *mouseconnectome.org* all provide images of the labeled sites. They allow browsing of injections by brain site. The *Allen* *mouse connectivity atlas* also exposes estimated densities of labeled sites. Textual statements derived from the *mouseconnectome.org* data will be available through *BAMS*.

The *temporal-lobe project* has chosen a standard brain map, and mapped all relevant tracing data onto it while entering the data into the system, thus relying on expert knowledge of the data collator.

Both *BAMS* and *CoCoMac* rely on some variant of ORT to bring injections and labeled sites into a common space. *BAMS*, in addition, has all regions mapped onto the Swanson rat atlas (Swanson, 1992) during the data entry phase. With the reimplementation and simplification of ORT in CoCoMac 2.0, computing a connectivity matrix has become a matter of seconds, and is available online at http://cocomac.g-node.org/axonal_projections. The implementation details are beyond the scope of this article, but we point out one important consideration. Translating information from one brain map to another relies on inter-map-relations, such as “A is identical to B” and “B is a subregion of C.” Such relations can also be nested: a subregion of a subregion is still a subregion, etc. The new ORT procedure starts with computing a matrix of all possible relations (identical, subregion, superregion, overlap) between all brain sites. In theory, this should result in *at most one* relation between any region pair. But for about 5% of the relations, conflicting versions exist. Wrong relations can have a detrimental effect on the connectivity matrix. CoCoMac 1.0 rather arbitrarily gave preference to identity relations. Modha and Singh (2010) produced a consistent spatial relations matrix by letting “is subregion of” prevail over “is identical to” statements. Both solutions neglect the fact that conflicts point to errors in the database or its literature sources. For CoCoMac 2.0 we have generated a list of core conflicts (Data Sheet 2 in Supplementary Material), which we are currently using to improve consistency across brain maps.

### COMPLETENESS AND LEVEL OF DETAIL

The *temporal-lobe* project defines a complete connectome as one that “contains all available knowledge,” and gets close to completeness for a selected set of brain regions. The 300 tracing studies contained in CoCoMac cover a substantial part of the available literature.

Another useful definition of completeness is: a connectome that covers the entire brain. A lower bound on completeness is that every brain region gets injected once, but given the variety of tracer characteristics, multiple injections are needed. The rodent brain workbench provides only six tracer injections, while the Allen mouse connectivity atlas systematically covers the entire brain. We have recently shown (Bezgin et al., 2012) that CoCoMac has full coverage, with between 2 and 80 injections (average 20) for the cortical regions of the Paxinos et al. (2000) atlas. If we require additional detail, such as laminar origin or termination patterns, the available data is reduced by a factor of four and covers most but not all regions.

The *temporal-lobe* project is a showcase of what level of detail can be obtained in a tract-tracing-based connectome. Connection strengths are allowed to vary within a brain region, by dividing it in nine subparcels. Within the subparcels, layer-specific termination patterns are indicated. Complete layer specificity cannot be obtained: anterograde tracers reveal layers of termination; retrograde tracers reveal layers of origin. But undetermined is whether there is full connectivity between these layers of origin and termination.

CoCoMac, *BAMS*, and most other projects distinguish between weak, moderate, and strong levels of labeled site density, but rarely do labeled sites get counted. Recent studies of Markov et al. (2011); Mikula et al. (2012) in which labeled cell bodies were counted showed variations by at least five orders of magnitude. This is essential data for computer simulations of brain tissue.

A limitation of all databases that rely on published tracing studies is that the textual descriptions of injection and labeled sites degrade the micro level accuracy of the tracing data to the macro level. A strategy to overcome this is to discard the legacy data and start a new, large-scale tracing effort that uses a standard atlas, records the stereotaxic location of injections and labeled sites, and registers all the individual brains to a common space. The *rodent brain workbench*, *mouseconnectome.org* and the *Allen mouse connectivity atlas* are such efforts. They expose the raw data in an online repository, to which automated approaches for counting labeled sites can be applied. Equivalent resources for macaque data are not available for two reasons: (1) ethical concerns restrict new experiments that duplicate legacy data,.and (2) the spatial location of brain regions has a much larger inter-subject variability, so that knowing the stereotaxic coordinate of an injection is not always more accurate than knowing its brain region.

### GRAPHICAL DISPLAY OF CONNECTIVITY

**FIGURE 2:**
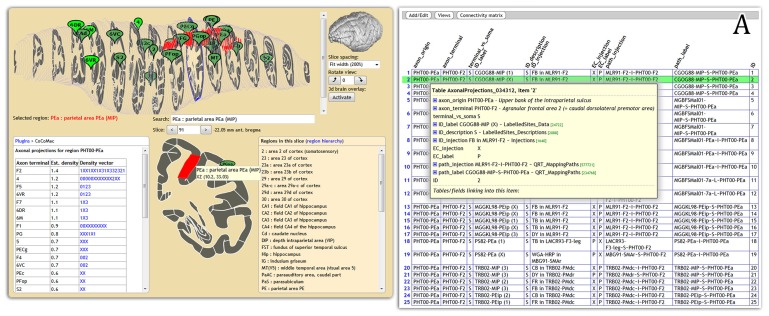
**Connectivity display using the Scalable Brain Atlas display engine (scalablebrainatlas.incf.org/cocomac), here displaying axonal projections that originate in parietal area PEa.** The insert **(A)** shows the result when clicking on a link in the connectivity table, each row can be expanded all the way down to the literature sources.

Display of connectivity is essential for disseminating results to non-experts. We are feeding CoCoMac connectivity results to the web-based atlas display engine at http://scalablebrainatlas.incf.org (SBA, Bakker et al., 2011, where users can query CoCoMac in a single mouse click. In turn, SBA redirects the user back to the CoCoMac website for a full traceback of the displayed connectivity. **Figure 2** demonstrates this procedure.

The *Connectome Toolkit* is another generic system for graphical display; this standalone, python-based package allows the creation of advanced scenes in which connectivity graphs and volumetric or surface based brain data can be blended, colored, and sliced.

The *temporal-lobe* project provides a poster-size interactive PDF document in which connections can be displayed by clicking on a region of interest. The document is well structured; but what is missing is a traceback to the original publications.

*NeuroVIISAS* finally has built-in rendering of connections on a 3D brain atlas. In addition, spiking activity generated by the built-in brain simulator makes the brain regions flash.

## FUTURE PERSPECTIVE

The future of tract-tracing connectomes hinges on three key questions:

1. Will tracing studies be obliterated by emerging new technologies?
2. How do we best deal with the existing, published data?
3. How do we best set up new experiments?

Among emerging technologies that compete with tract-tracing are diffusion based MRI, polarized light imaging (Axer et al., 2011), and serial block-face scanning electron microscopy (SBFSEM). They share as a big advantage that a complete connectome can be derived from a single brain, thus eliminating the loss of resolution that occurs when data from different brains is combined. Diffusion MRI plays a key role in the Human Connectome Project (Marcus et al., 2011), where invasive techniques are not an option. This project is unique in that its primary aim is not to publish a connectome, but rather make the best possible data and analysis tools publicly available that will allow others to do so. Also applicable to humans is the PLI method, which uses polarized light to measure the orientation of myelinated fibers in thin post-mortem brain slices. This is done at microscopic resolution in the in-plane dimensions. The challenge lies in the across-slice dimension, where slices have to be realigned after cutting and non-uniform shrinking. This same problem initially hampered electron microscopy approaches, but with the introduction of serial block-face scanning it became possible to obtain resolutions below 50 nm in *all* dimensions (Denk and Horstmann, 2004). Such resolutions may become available for an entire mouse brain (Mikula et al., 2012) in about 10 years, and promise to reveal full axonal and dendritic trees – provided that automated techniques become available that digest petabytes of data without human intervention.

For the coming decades, tract-tracing will remain the gold standard (Sporns, 2010), because it provides (1) unequivocal proof for the existence of long-range connections; (2) directionality; (3) layer specificity, and (4) sufficient detail to estimate quantitative connection strengths. In addition, recently developed tracer substances can reveal connections that are (5) cell type-specific and (6) part of local, multisynaptic circuits (Vercelli et al., 2000).

The question on how to best deal with existing, published tracing data has been largely addressed in this manuscript. One further aspect is to provide large-scale brain simulators with realistic parameter estimates for layer- and cell type-specific connectivity. This requires an integrative approach that combines tract-tracing studies with cell reconstructions and multi cell patch clamp studies (Potjans and Diesmann, 2011).

For the final question on how to design new tract-tracing studies, the *rodent brain workbench*, *mouse connectome, *and *Allen mouse connectivity atlas* pave the way: register sliced individual brains to a standard brain, and provide public access to the raw tracing images. The approach has four remaining challenges: (1) providing access to large data sets is not financially rewarding; (2) brain parcellations needed to convert imaging data to textual statements are often copyrighted; (3) automated extraction of connectivity data is a daunting task, highly dependent on the *meta data*; and (4) especially for primates, ethical guidelines require that legacy data are *not discarded*. The International Neuroinformatics Coordinating Facility (INCF^6^) aims to relief these challenges by promoting standards, open access, and developing a global federated data space. The present connectome projects each have unique properties, which are best combined by supporting a common interface and data format. That would make it possible to cross-check connectivity data from multiple databases, in the same way as CoCoMac already uses redundant inter-map-relations to improve consistency across brain parcellations.

## Conflict of Interest Statement

The authors declare that the research was conducted in the absence of any commercial or financial relationships that could be construed as a potential conflict of interest.

## Footnotes

1 mouseconnectome.org

2 http://connectivity.brain-map.org/

3 http://www.mysql.com

4 http://www.php.net

5 http://cocomac.g-node.org/search_wizard

6 www.incf.org
